# Progress in Surgery

**Published:** 1902-06-07

**Authors:** 


### Progress in Surgery.

### OTOLOGY.

**The Edelmann-Galton Whistle.**—H. Macnaughton Jones [1] has written a description of the above instrument. He points out that it is obviously very important for otologists to possess a thoroughly reliable instrument for testing the perceptive power of the ear for high tones, especially for use side by side with the tuning-fork in attempting to distinguish between the deafness due to middle-ear disease and that due to disease of the labyrinth or auditory nerve. The Galton whistle, as most recently modified by Professor von Edelmann, is clearly a much more scientific and accurately gauged instrument than those which have hitherto been in general use. Professor Edelmann gives an account of his whistle in the "Zeitschrift für Ohrenheilkunde," Band xxxvi., and there shows that it was by following A. Schwendt (Basel) and using Kundt's dust-figures for the determination of the wave-length and the number of vibrations per second of each note produced by the whistles that he was able to make important improvements in their construction and graduation. The dust-figures are produced in the following way. A well-cleaned and dried glass-tube is closed at one end with a cork, or if narrow with sealing-wax, and a small quantity of dry lycopodium powder introduced. The powder is first shaken down to the corked end, then, by shaking the tube with the open end inclined obliquely downward, and by gently tapping it with a slender piece of wood, the powder is spread along the tube in an even band-like layer. The latter is then held by a clamp, or if small by a stick of sealing-wax fixed to a table, and slightly turned, so that the layer of powder takes a position slightly to one side. The whistle is sounded with its mouth opposite the open end of the tube. The air in the tube vibrates in unison with the whistle, and the powder is shaken most and falls to the lowest points where the air-movements are greatest, but remains unmoved where the nodes of the sound-waves are situated. Thus a festoon-like dust-figure is produced. The figures can be beautifully seen against a dark ground of black paper or cloth. The distance between two nodes can be measured with compasses, and that distance equals a half wave-length. From this the number of vibrations per second is easily calculated by dividing the velocity of sound by the wave-length, *i.e.*, the Number of waves $= \frac{\text{Velocity of sound (in millimetres)}}{\text{Length of wave (in millimetres)}}$
Edelmann's whistle consists of an upper cylindrical part, the pipe; and a lower cylindrical part, the mouthpiece; and the two are fixed by a metal handle, the one vertically above the other. Air is propelled through the mouthpiece from a small rubber ball. By the dust-figures it is easily shown that clear notes are largely dependent for their production upon the distance of the mouthpiece from the pipe; therefore this distance can be varied in the instrument by a graduated screw. The length of the pipe containing the column of air which has to vibrate can also be varied in a very accurate way with another screw which moves a piston in the cylinder. By adjusting the readings of the two screws mentioned in accordance with a table provided with each instrument, various notes are produced varying in their number of vibrations per second between 3,480 and about 50,000. Some people are found with this instrument to be able to hear tones of 50,000 or even more vibrations per second. "When formerly the highest audible tones were supposed to have been reached," says Edelmann, "it was more probable that the pipe did not sound than that the ear was unable to perceive it."

**The Treatment of Chronic Non-suppurative Middle-ear Disease.**—Chalmers Watson (Edin.) [2] has published some results he has obtained from the treatment of the above affection with a preparation of bone-marrow, and these results seem sufficiently good to merit attention. Watson's theory is that bone-marrow produces an internal secretion of vital importance, and that the substance is a powerful prophylactic against the injurious action of various saprophytic fungi which are present in health in various tissues. Introductory experiments in middle-ear catarrh were made to test the effect of olive oil, which might be expected to act as a mechanical lubricant, and of a combination of glycerine and rectified spirits, which has a stimulating effect on the circulation in the membrane tympani and middle ear. The watch-test of hearing was soon abandoned as of no practical value, and Watson's experience with the whisper-test showed that there was no definite relation between the perception for whispering and that for conversation in an ordinary tone. He found also that a low-pitched voice used as a test showed often an improvement in the hearing distance of a few inches only, when an ordinary voice at medium pitch showed a gain of 7 or 8 feet. The writer points out that due attention must be given to the patient's own reports as to his hearing capacity and to the statements of members of the same household. The results of 20 cases submitted to this treatment are summarised. With the exception of two all the patients had been treated by well-known experts and had been given to understand that their condition was practically hopeless. Full notes are given of two cases. The first patient was a young woman of 21, who had had middle-ear trouble for three years. First instillations of warm olive oil into external meatus were made daily for five weeks. The results of this treatment were practically *nil*. Then instillations of the glycerine and spirit mixture were tried for about nine weeks. There was considerable improvement. Relapse followed on cessation of the treatment. The preparation was used again and again: there was improvement, but it was of only transient duration. After stopping this treatment the hearing became as bad as ever. Then the treatment with "myelocene" was begun in conjunction with spirit and glycerine. A gratifying increase of hearing power soon followed and the patient became much brighter and more alert-looking. Some deterioration of hearing was shown on stopping the treatment, but improvement again followed its resumption. After a three months' interval without treatment, though there was some degree of relapse, the patient could still hear better than before the treatment had been given. In the second case, of which full details are recorded, the improvement gained was fully maintained after cessation of the treatment. Of 15 cases treated with myelocene 11 showed improvement comparable to those detailed above, and only two cases were uninfluenced for good. Myelocene is prepared by extracting healthy marrow with ether, evaporating the solution, and then rubbing up the fat with chloretone for preservative purposes.

[1] Edin. Med. Jour., April 1902, p. 349. [2] Brit. Med. Jour., March 22, 1902, p. 699.

